# Modifiable and Non-modifiable Factors Associated with Low Awareness of Hypertension Treatment in Indonesia: A Cross-Sectional Population-Based National Survey

**DOI:** 10.5334/gh.1143

**Published:** 2022-08-16

**Authors:** Qisty A. Khoiry, Sofa D. Alfian, Rizky Abdulah

**Affiliations:** 1 Department of Pharmacology and Clinical Pharmacy, Faculty of Pharmacy, Universitas Padjadjaran, Jatinangor, Indonesia; 2 Center of Excellence in Higher Education for Pharmaceutical Care Innovation, Universitas Padjadjaran, Jatinangor, Indonesia

**Keywords:** Awareness, Hypertension, Medication, Treatment

## Abstract

**Introduction:**

Low awareness about hypertension treatment is recognized as a significant cause of treatment failure. Therefore, identifying its underlying factors is essential for developing effective intervention strategies. This study aims to identify the modifiable and non-modifiable factors associated with low awareness about hypertension treatment.

**Method:**

This national, cross-sectional, population-based survey used publicly available data from the Indonesian Family Life Survey (IFLS-5) for 2014 among respondents with hypertension aged ≥15 years. Depression and insomnia, as modifiable factors, were assessed using the Centre for Epidemiologic Studies—Depression (CES-D) and the Patient-Recorded Outcomes Measurement Information System (PROMIS) questionnaire, respectively. Non-modifiable factors, such as sociodemographic information, were obtained from self-reported data. Logistic regression analysis was used to assess the association between these factors and low awareness about hypertension treatment. Odds ratios (ORs) with 95% confidence intervals (CIs) were reported.

**Result:**

The study recruited 7,920 respondents, the majority of whom were female (53.8%) and aged <60 years (71.1%). The prevalence of low awareness of hypertension treatment was 87.1% (51.8% in women and 48.2% in men). Being an elderly (OR: 1.60, 95%CI 1.36–1.88), being irregularly blood pressure control (OR: 4.40, 95% CI 3.78–5.13), having depressive symptoms (OR: 1.35, 95% CI 1.12–1.62), having insomnia (OR: 1.31, 95% CI 1.11–1.53), and having low satisfaction with health care (OR: 1.28, 95% CI 1.08–1.51) were associated with low awareness of hypertension treatment. Surprisingly, respondents with strong religiosity (OR: 1.62; 95% CI 1.25–2.09) were more likely to display low awareness of hypertension treatment.

**Conclusion:**

The main factors associated with low awareness of hypertension treatment are modifiable. Thus, health care professionals should integrate more patient-specific factors when designing tailored interventions.

## Introduction

Hypertension is an increasing part of the global burden of disease, which is more predominant in low- and middle-income countries (LMICs) [1]. The current global trends in hypertension has increased from 650 million to 1.28 billion over the last three decades [2]. A population-based study on more than 90 countries revealed that the burden of hypertension has increased by 7.7% in LMICs but decreased by 2.6% in high-income countries (HICs) [3]. Globally, 8.5 million deaths were associated with uncontrolled high blood pressure due to unawareness of this condition [4]. In fact, awareness of hypertension treatment is reportedly lower in LMICs compared with other countries [5]. In 2017, Indonesia reported 91.3 million people with hypertension, which was responsible for 35% of all deaths [6,7].

Uncontrolled blood pressure is predominantly frequent in LMICs, where health awareness is the weakest and mortality rates due to cardiovascular disease are the highest [8]. According to Indonesia Basic Health Research 2018, only 37% of Indonesians with hypertension currently have obtained control of their blood pressures, whereas 45.6% exhibited low adherence to hypertension treatment [7]. Low awareness of medication is reported to be associated with medication non-adherence [9,10], which may lead to poorly controlled hypertension, high risks of complication, and increased health care costs [11,12,13]. Therefore, low awareness of hypertension treatment should be addressed due to the lack of symptoms of the disease.

Previous studies have reported several factors that may be associated with awareness of hypertension treatment [14,15,16,17]. However, such studies have mainly explored non-modifiable factors. A previous study conducted in LMICs revealed that insurance status and income were significantly associated with awareness of treatment of hypertension [18]. Moreover, low levels of education were associated with low rates of awareness about hypertension treatment in LMICs but not in HICs [5]. However, the association of modifiable factors, such as satisfaction with health care, blood pressure control status, depressive symptoms, and insomnia, to awareness of hypertension treatment in Indonesia remains unclear.

The Indonesian Family Life Survey (IFLS) is a longitudinal socioeconomic and health survey in Indonesia, which represents approximately 83% of the Indonesian population [19]. Several studies that utilized IFLS-4 or IFLS-5 demonstrated the association of non-modifiable factors, such as socioeconomic status [20] and psychosocial risk [17], to hypertension in Indonesia. However, a clear evidence of which factors or foci are required to improve medication awareness of hypertension treatment in Indonesia remains lacking. Thus, identifying its underlying factors is essential to developing effective strategies for intervention. This study aims to identify modifiable and non-modifiable factors associated with low awareness of hypertension treatment in Indonesia.

## Methods

### Study design

This study used the national longitudinal and cross-sectional data of the fifth wave of the IFLS-5, which were collected from 2014 to 2015 and were publicly available since 2016. IFLS-5 used a multistage stratified sampling design that represents 83% of the Indonesian population conducted in 13 provinces, which are four provinces on Sumatra (North Sumatra, West Sumatra, South Sumatra, and Lampung), all five of the Javanese provinces (DKI Jakarta, West Java, Central Java, DI Yogyakarta, and East Java), and four provinces covering the remaining major island groups (Bali, West Nusa Tenggara, South Kalimantan, and South Sulawesi) with a response rate of more than 90% [19]. The IFLS collected extensive measures of basic sociodemographic, certain economic characteristics of all household members, and health status, including self-reported measures of general health status, symptoms, pain, and biomarker measurements of health. The ethical review boards have approved the IFLS of Research and Development (RAND) Corporation and Gadjah Mada University. Written informed consent was obtained from all respondents before data collection [19].

### Study population

The total number of IFLS-5 subjects was 34,464 (aged 0 to >80 years). Data were obtained from subjects who were at least 15 years of age at the completion of the survey. Subjects with available data on blood pressure, depressive symptoms, and sleeping quality were included. Subjects with missing values for outcome variables were excluded.

### Outcome measure

Subjects with hypertension were defined as having a blood pressure ≥140/90 mm Hg [21]. Blood pressure was measured three times at home by specially trained nurses using an Omron sphygmomanometer (HEM-7203) as the subjects were seated. The first measurement was taken at the beginning of the interview with two subsequent measures taken during the interview [19]. The average of the three measurements was used for analysis.

Awareness of treatment was defined by responses to the following question: *In order to manage your hypertension, are you currently taking prescribed medication weekly?* Those who answered yes were considered to have a high awareness of hypertension treatment, whereas the opposite is true for those who responded with no. Awareness of hypertension treatment refers to those who were aware of their condition, aware they need to have treatment, and receiving treatment for their condition [22,23]. It is relatively different from adherence, which was defined as the process by which patients take their medications as prescribed, composed of initiation (when the first dose is started), implementation (the actual dose of the patient from the start to the last dose), and discontinuation (end of treatment) [24].

### Potential factors associated with low awareness of hypertension treatment

The potential factors associated with low awareness of hypertension treatment were classified as modifiable and non-modifiable factors. Depressive symptoms, having insomnia, blood pressure control status, happiness status, having visited health care services, and satisfaction with health care were categorized into modifiable factors. Alternatively, non-modifiable factors consisted of gender, age, marital status, level of education, residency, coverage of health insurance, economic status, and religiosity.

A self-reported Center for Epidemiologic Studies Depression (CES-D) scale was used to measure depressive symptoms [19]. CES-D contains 10 questions to assess the feelings of subjects, which are highly correlated with depressive symptoms [25]. Eight questions were focused on negative symptoms (e.g., *I was bothered by things that usually do not bother me*), whereas the two other questions assessed the positive symptoms of depression (e.g., *I felt hopeful about the future*). The subjects indicated the frequency that each item applied to them in the past week using a four-point Likert-type scale (0 = rarely or none of the time; 1 = some or little of the time; 2 = moderately or much of the time; 3 = most or almost all the time). The final score is calculated by summing all items after reversing the positive mood items. A subject with a total score equal to or above 10 is considered to have symptoms of depression [26]. The CES-D questionnaire was translated into Indonesian (forward translation), where two independently hired translators re-translated it into English (back translation) [19].

Insomnia was assessed using 10 questions from PROMIS [19]. Five items each were used to assess sleep quality and sleep impairment in the past week [27]. Each item was rated using a five-point Likert-type scale (0 = never/not at all; 1 = a little bit; 2 = somewhat; 3 = quite a bit; 4 = always/very much). Insomnia was defined as total scores of ≥21–40 [28]. The PROMIS questionnaire was translated into Indonesian (forward translation), where two independently hired translators re-translated it into English (back translation) [19].

Blood pressure control status was categorized as regular and irregular dependent on the subject’s response to the question, *How regularly do you have your blood pressure checked?* Happiness status was assessed using the question, *Taken together, how you would say things are these days? Would you say you are very happy, happy, unhappy, or very unhappy?* Those who answered *very happy* or *happy* were categorized as happy, whereas those who answered *unhappy* or *very unhappy* were categorized as unhappy [29]. Satisfaction with health care was assessed using the question, *Concerning your health care, which of the following is true: it is less than adequate for my needs; it is just adequate for my needs; it is more than adequate for my needs?* Low satisfaction with health care is equivalent to the response *it is just less than adequate for my needs*, whereas high satisfaction with health care is indicated by *it is just adequate for my needs* or *it is more than adequate for my needs* [29].

Sociodemographic factors were gender (men, women), age at the completion of the survey (less than 60 years for the non-elderly and more than 60 years for the elderly), marital status (currently unmarried and currently married), level of education (no education, elementary school, junior high school, senior high school, and university), residency (rural and urban), coverage of health insurance (no and yes), economic status (poor and non-poor), and religiosity (religious, non-religious). The total income of the family was used to measure economic status over the past 12 months in rupiah divided by family size (per capita income). Capita income was categorized per quintile. Religiosity was assessed using the question *How religious are you?* Those who answered *very religious* or *religious* were categorized as religious, whereas those who answered *somewhat religious* or *not religious* were considered as non-religious [29]. The subject was asked whether they visited any health care services one month prior to the survey to assess health care visit status (yes and no).

To determine whether subjects have comorbid disease status, subjects were asked the question, *Has a doctor/paramedic/nurse/midwife ever told you that you had the following chronic conditions of diseases?* with response options: hypertension, diabetes, tuberculosis, asthma and other chronic lung diseases, cardiac diseases(heart attack/coronary heart disease/angina or other heart diseases), liver diseases, stroke, cancer or malignancies, arthritis/rheumatism, uric acid/gout, depression, vision and hearing abnormalities [29]. Subjects who answered only hypertension were categorized as having no comorbidity, while subjects who answered hypertension and one to three other chronic disease were then categorized as having one to three comorbidities, and subjects who had more than four comorbidities were categorized as having more than four comorbidities.

### Statistical analysis

Descriptive statistics were used to summarize the characteristics of the subjects. Awareness of hypertension treatment was estimated for each age group and gender. A chi-square test was performed to assess the univariate association between the characteristics of the subjects and the outcome. We conducted complete case analyses because the missing data were small. The potential factors found associated with the outcome at a significance level of *p* < 0.25 in the univariate analyses were included in the initial multivariate model. Multivariate logistic regression was performed to obtain the odds ratio (OR) with a 95% confidence interval (95% CI) with manual backward elimination. The *p*-values were set at 0.05 for factors included in the final model. Subgroup analyses by number of comorbidities was performed in no comorbid, having one to three comorbidities, and having more than four comorbidities groups. The goodness-of-fit statistic was assessed using the Hosmer-Lemeshow test. Pseudo R-squared value, which is the default value reported by Stata, is obtained as an equivalent to the R-squared reported in the linear regression. R-squared is a number that ranges from 0 to 1, which indicates the extent to which the combination of independent variables simultaneously influences the value of the dependent variable [30]. All statistical analyses were performed using Stata software version 14.0 for Windows.

## Results

A total sample of 7,920 subjects without missing data on awareness of hypertension treatment was included in this study. The majority were female (53.8%) and aged <60 years (71.1%; Table 1). A high proportion of subjects displayed poor blood pressure control (80.5%), although most of them (90.8%) visited health care facilities in the last month. Approximately half of subjects were covered by health insurance (50.4%), resided in rural areas (57.6%), and graduated from elementary school (41.2%). Out of the total participants, 20.4% experienced depression, whereas 46.3% have insomnia.

**Table 1:** Baseline Characteristic of Study Population (*N* = 7,920).


CHARACTERISTIC	NUMBER	%

**Awareness of Hypertension Treatment**		

Low Awareness	6,895	87.1

High Awareness	1,025	12.9

**Comorbidity Status**		

No Comorbid	5,134	67.1

1–3 Comorbidities	2,441	31.8

≥4 Comorbidities	81	1.1

Missing	264	—

**Gender**		

Women	4,263	53.8

Men	3,655	46.2

Missing	2	—

**Age**		

Non-elderly [<60 years old]	5,627	71.1

Elderly [≥60 years old]	2,291	28.9

Missing	2	—

**Education Level**		

No Education	1,021	13.3

Elementary School	3,173	41.2

Junior High School	1,099	14.3

Senior High School	200	2.6

University	2,208	28.7

Missing	219	—

**Marital Status**		

Not Currently Married	2,138	27.0

Currently Married	5,780	73.0

Missing	2	—

**Blood Pressure Control Status**		

Irregularly	4971	80.5

Regularly	1,201	19.5

Missing	1,748	—

**Economic Status**		

Quintile 1	1,605	20.3

Quintile 2	1,661	21.0

Quintile 3	1,498	18.9

Quintile 4	1,595	20.1

Quintile 5	1559	19.7

Missing	2	—

**Health Care Satisfaction**		

Low	1,837	25.3

High	5,411	74.7

Missing	672	—

**Happiness Status**		

Unhappy	857	11.8

Happy	6,391	88.2

Missing	672	—

**Insomnia**		

Yes	3,353	46.3

No	3,895	53.7

Missing	672	—

**Depressive Symptoms Status**		

Not Depressed	5,768	79.6

Depressed	1,480	20.4

Missing	672	—

**Residency**		

Rural	4,460	57.6

Urban	3,289	42.4

Missing	171	—

**Health Care Visit Status**		

No	1,548	90.8

Yes	156	9.2

Missing	6,216	—

**Coverage of Health Insurance**		

No	2,105	49.6

Yes	2,141	50.4

Missing	3,674	—

**Religiosity**		

Not Religious	1,248	17.2

Religious	6,002	82.8

Missing	670	—


The prevalence of low awareness of hypertension treatment was 87.1% (51.8% in women and 48.2% in men). Level of education, marital status, economic status, and happiness status were negatively associated with low awareness of hypertension treatment (Table 2). Gender, age, blood pressure control status, satisfaction with health care, insomnia, depressive symptoms status, residency, health care visit status, coverage of health insurance, and religiosity were selected as potential factors associated with low awareness of hypertension treatment based on univariate analyses.

**Table 2:** Univariate Associations with Awareness of Hypertension Treatment.


CHARACTERISTIC	LOW AWARENESS OF HYPERTENSION TREATMENT		HIGH AWARENESS OF HYPERTENSION TREATMENT		P-VALUE
	
	*N*	%	*N*	%	

**Gender**					0.000*

Women	3,568	51.8	695	67.9	

Men	3,326	48.2	329	32.1	

**Comorbidity Status**					0.000*

No Comorbid	4,865	70.5	269	35.3	

1–3 Comorbidities	1,977	28.7	464	61	

≥4 Comorbidities	53	0.8	28	3.7	

**Age**					0.000*

Non-elderly [<60 years old]	5,007	72.6	620	60.5	

Elderly [≥60 years old]	1,887	27.4	404	39.5	

**Education Level**					0.368

No Education	876	13.1	145	14.5	

Elementary School	2,761	41.2	412	41.3	

Junior High School	960	14.3	139	13.9	

Senior High School	182	2.7	18	1.8	

University	1,924	28.7	284	28.5	

**Marital Status**					0.910

Not Currently Married	1,860	26.9	278	27.2	

Currently Married	5,034	73.1	746	72.8	

**Economic Status**					0.251

Quintile 1	1,396	20.3	209	20.4	

Quintile 2	1,462	21.2	199	19.4	

Quintile 3	1,309	19.0	189	18.5	

Quintile 4	1,395	20.2	200	19.5	

Quintile 5	1,332	19.3	227	22.2	

**Residency**					0.000*

Rural	2,924	42.4	660	64.4	

Urban	3,971	57.6	365	35.6	

**Health Care Visit Status**					0.000*

No	1,046	88.6	502	96.0	

Yes	135	11.4	21	4.0	

**Coverage of Health Insurance**					0.000*

No	1,892	51.0	320	60.0	

Yes	1,821	49.0	213	40.0	

**Religiosity**					0.000*

Not Religious	1,139	18.0	107	11.7	

Religious	5,191	82	811	88.3	

**Blood Pressure Control Status**					0.000*

Irregularly	5,328	84.2	514	56.1	

Regularly	1,001	15.8	403	43.9	

**Health Care Satisfaction**					0.000*

Low	1,553	24.5	284	30.9	

High	4,777	75.5	634	69.1	

**Happiness Status**					0.210

Unhappy	737	11.6	120	13.1	

Happy	5,593	88.4	798	86.9	

**Insomnia**					0.000*

Yes	2,858	45.2	423	46.1	

No	3,472	54.8	495	53.9	

**Depressive Symptoms Status**					0.000*

Not Depressed	5,091	80.4	677	73.7	

Depressed	1,239	19.6	241	26.3	


* Included in initial multivariate model.

In the multivariate model, the elderly (OR: 1.60; 95% CI: 1.36–1.88) and subjects with low satisfaction with health care (OR: 1.28; 95% CI: 1.08–1.51) were associated with low awareness of hypertension treatment. Interestingly, subjects with high religiosity were likely to exhibit low awareness of hypertension treatment (OR: 1.55; 95% CI: 1.24–1.94). Modifiable factors associated with low awareness of hypertension treatment were having depressed symptoms (OR: 1.35; 95% CI: 1.12–1.62), having insomnia (OR: 1.31; 95% CI: 1.11–1.53), and blood pressure control status (OR: 4.40; 95% CI: 3.78–5.13). Irregular blood pressure control exhibited the highest odds of low awareness of hypertension treatment (Table 3). The goodness-of-fit p-value of the model was 0.271 with a pseudo-R-squared value of 8.74%. Overall, modifiable and non-modifiable factors that were associated with low awareness of hypertension treatment were relatively similar in subgroup analysis, except that insomnia and depressive symptom status were not seen as risk factors for low awareness of hypertension treatment among subjects with more than four comorbidities (Table 3).

**Table 3:** Association between Modifiable and Non-modifiable Factors with Low Awareness of Hypertension Treatment with Subgroup Analysis Result.


CHARACTERISTIC	OVERALL^a^		SUBGROUP ANALYSIS: NO COMORBIDITIES		SUBGROUP ANALYSIS: 1–3 COMORBIDITIES		SUBGROUP ANALYSIS: ≥4 COMORBIDITIES	
			
	OR [95% CI]^b^	P-VALUE	OR [95% CI]	P-VALUE	OR [95% CI]	p-value	OR [95% CI]	p-value

Age								

Non-elderly(<60 years old)	Reference		Reference		Reference		Reference	

Elderly (≥60 years old)	1.60 [1.36–1.88]	0.000	1.72 [1.30–2.29]	0.000	1.82 [1.42–2.32]	0.000	1.34 [0.39–4.61]	0.641*

Blood Pressure Control Status								

Regularly	Reference		Reference		Reference		Reference	

Irregularly	4.40 [3.78–5.13]	0.000	3.47 [2.63–4.60]	0.000	4.48 [3.54–5.69]	0.000	2.14 [0.64–7.16]	0.219*

Depressive Symptoms Status								

Not Depressed	Reference		Reference		Reference		Reference	

Depressed	1.35 [1.12–1.62]	0.002	1.04 [0.73–1.48]	0.821*	1.37 [1.04–1.82]	0.028	0.50 [0.10–2.47]	0.398*

Insomnia								

No	Reference		Reference		Reference		Reference	

Yes	1.31 [1.11–1.53]	0.001	1.46 [1.02–2.09]	0.041	1.19 [0.86–1.66]	0.293*	1.09 [0.21–5.74]	0.916*

Health Care Satisfaction								

High	Reference		Reference		Reference		Reference	

Low	1.28 [1.08–1.51]	0.003	1.27 [0.94–1.71]	0.114*	1.14 [0.88–1.47]	0.324*	0.60 [0.16–2.21]	0.442*

Religiosity								

Not Religious	Reference		Reference		Reference		Reference	

Religious	1.55 [1.24–1.94]	0.000	1.75 [1.14–2.68]	0.011	1.56 [1.09–2.22]	0.016	2.29 [0.38–13.70]	0.036*


^a^ Goodness-of-fit p-value: 0.271; pseudo-R-squared: 8.74%. ^b^ Final multivariate model. * Not significant.

## Discussion

Among the 7,920 respondents, 87.1% reported low awareness of hypertension treatment. Age and religiosity are non-modifiable factors associated with low awareness of hypertension treatment. Meanwhile, the modifiable factors are blood pressure control status, satisfaction with health care, depressive symptoms, and insomnia. Medication awareness is critical in providing accurate information about the treatment undertaken to improve medication adherence [31]. Patients with low awareness of treatment tend to be non-adherent to treatment for chronic diseases [32]. Medication awareness is one of the five broad categories of treatment-related problems in a patient perspective that have potential implications for therapeutic success as they create barriers to medication adherence [33]. However, low adherence to treatment in chronic disease is associated with poor disease control, increased costs, increased hospitalization rates, decreased quality of life, and increased mortality [34,35,36,37].

We observed that the elderly subjects were associated with low awareness of hypertension treatment, which can be partially explained by several possible barriers, such as frailty, multiple comorbidities, polypharmacy, and cognitive impairment [38]. Surprisingly, religiosity indicated a significant association with low awareness of hypertension treatment. A possible explanation may be that people foresee and surrender healing outcomes from God. Thus, they would risk not being aware of taking medications [39,40]. In addition, previous studies reported that religiosity could increase the likelihood of using complementary and alternative medical (CAM) practices, such as herbal medicines [41,42]. The high prevalence of CAM among patients in Ethiopia with hypertension is associated with low awareness of health care, which potentially leads to ineffective hypertension management [43]. Another finding from a previous study reported decreased adherence to prescribed medication due to CAM use in patients with type 2 diabetes [44].

We further observed that blood pressure control status, satisfaction with health care, depressive symptoms, and insomnia are associated with low hypertension awareness. Subjects who irregularly control their blood pressure have a risk of low awareness of hypertension treatment. A possible explanation for this finding may be that the inadequate understanding of therapeutic goals influences their awareness of hypertension treatment [45]. Affordability and unreliable devices are the two main reasons why patients do not check their blood pressure regularly [46]. Individual medical education is the most critical aspect of blood pressure regulation [47]. The fundamental method for hypertension control is to make people more aware of it, treat it, and maintain it under control [48]. The problem of achievement of target blood pressure consists of patient-related barrier, physician-related barrier, and health care system-related barrier, so that a team-based approach to blood pressure control that involves nurses, pharmacists, and physician assistants should be emphasized [49]. People with high blood pressure can benefit from antihypertensive medication, while complications and aging make it less probable to achieve the desired blood pressure [47].

We also observed that patients with low satisfaction with health care were likely to display low awareness of hypertension treatment. Previous studies revealed that satisfaction with health care was associated with adherence to hypertension treatment [50,51]. Subjects with better satisfaction with health care may increase their awareness of hypertension treatment. Furthermore, having a depressive status was associated with low awareness of hypertension treatment, which can be partially explained by the deterioration of high-level cognitive functions, specifically attention, awareness, and memory, among subjects with depressive symptoms [52]. At the same time, having depressive disorders has been associated with hypertension [53,54,55]. A systematic review and meta-analysis demonstrated that the risk for cardiovascular diseases in subjects with depressive symptoms is approximately two times higher than those in non-depressed people [56]. Similar results were observed in subjects with insomnia. A possible explanation is that the severe decrease in sleep hours is related to cognitive function, which leads to the lack of awareness of treatment. Prior studies have reported a significant relationship between sleep duration and cognitive function [57,58]. This finding indicates the need to screen for sleep impairment and to maintain regular sleep patterns.

Hypertension frequently coexists with diabetes, hyperlipidemia, or other metabolic syndromes [59]. Patients with these comorbidities are more likely to require combination therapy, which needs more medications. The result of subgroup analysis showed that both subgroups had relatively similar modifiable and non-modifiable factors that were associated with low awareness of hypertension treatment. Therefore, health care providers need to pay more attention to patients with low awareness regardless of their number of comorbidities.

In summary, the majority of sociodemographics as non-modifiable factors were not associated with low awareness of hypertension treatment. Sociodemographics such as marital status may be extremely general for predicting the medication awareness of subjects. No association was found between gender and low awareness of hypertension, as reported in a previous study [60]. In general, gender is seemingly a less meaningful factor associated with low awareness of hypertension treatment. Moreover, the level of education is not associated with awareness, which is similar to the findings of previous studies [61,62]. Thus, health literacy may be more important than level of education [62]. By contrast, another study indicated that low levels of education were associated with low awareness of hypertension in LMICs [5]. We observed that residency was not associated with low awareness of hypertension treatment. This finding is in contrast with that of a previous study [63], which found an association between people living in urban areas and high awareness of hypertension treatment. Population in urban areas has exhibited better treatment-seeking behavior and has easy access to health care [63]. Notably, the current study observed no association between visits to health care facilities with low awareness of hypertension treatment. In contrast, a study using IFLS-4 indicated that the number of visits to any health care facility helped to increase awareness about hypertension [64]. Furthermore, no association was found between happiness status and low awareness of hypertension treatment. This result is consistent with data obtained in a cohort study with a 31-year follow-up in London, which revealed the lack of robust associations between life satisfaction and incident hypertension [65]. One unexpected finding is that health insurance coverage was not associated with low awareness of hypertension treatment. However, one could expect that the limited subjects had health coverage in 2014 since the Indonesian National Health Insurance System began in 2014. Moreover, other studies reported on the positive effect of health insurance coverage due to the reduced cost of receiving health care services [66,67].

**Figure 1:**
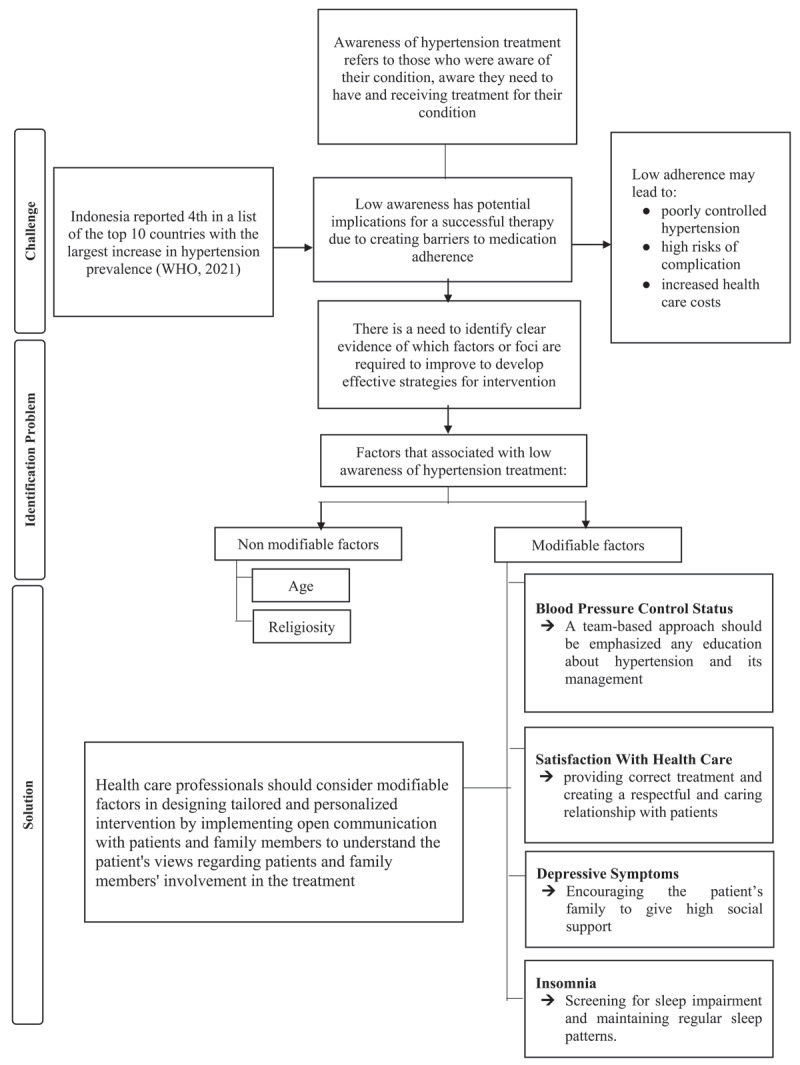
Problems, Challenges, and Solutions to Increase Low awareness of Hypertension Treatment in Indonesia.

The current finding suggests that the majority of non-modifiable factors may be insufficient to address the low awareness of hypertension treatment. Thus, health care professionals should consider modifiable factors in designing tailored and personalized interventions by implementing open communication to educate and to understand patients’ views regarding patients’ and family members’ involvement in the treatment to improve patients’ health outcomes [68]. Education and counseling are effective in terms of behavioral changes in patients to clarify misperceptions and improve the awareness of hypertension treatment [69]. Furthermore, health care professionals should to regularly check blood pressure and consider patient satisfaction by providing correct treatment and creating a respectful and caring relationship with patients by sharing the decision-making process [70]. The current findings may serve as reference for health professionals focusing on factors related to low awareness of hypertension treatment that should be addressed (Figure 1).

The strength of this study is that we used IFLS data, which represents 83% of the Indonesian population with a small attrition rate (6%). However, we acknowledge certain limitations related to methodological issues. First, the study was unable to provide causal inferences regarding the association between modifiable and non-modifiable factors related to low awareness of hypertension treatment due to the cross-sectional design. Second, we used complete case analysis, which may reduce statistical power due to the sample size, which can produce biased estimates, leading to overestimating or underestimating conclusions. Third, in this analysis, we included only subjects with a blood pressure of more than 140/90 mmHg, which may lead to overestimating low awareness of hypertension treatment since subjects with controlled blood pressure (blood pressure <140/90 mmHg) are not included. Fourth, the overall association of our model was relatively low, which indicated the presence of other unmeasured factors that may influence the low awareness of hypertension treatment, such as education about hypertension, origins of subjects, local tradition, number of medication, medication beliefs [71], comorbid diseases [72], or healthy lifestyle [73].

## Conclusions

The main factors associated with low awareness of hypertension treatment are modifiable. Thus, health care professionals should integrate more patient-specific factors when designing tailored interventions.
